# Three years of tele-emergency medicine with mobile on-site audio-video streaming in lower Saxony, Germany – descriptive results of a longitudinal secondary data analysis

**DOI:** 10.1186/s12873-025-01286-w

**Published:** 2025-07-15

**Authors:** Johanna Sophie Lubasch, Insa Seeger, Thomas Marian, Tobias Steffen, Friederike Schlingloff

**Affiliations:** 1 Research Network Emergency and Intensive Care Medicine, School of Medicine and Health Sciences, Carl von Ossietzky Universität Oldenburg, Ammerländer Heerstraße 114-118, Oldenburg, 26129 Deutschland; 2 District of Goslar, Emergency Rescue Services, Ottostraße 1, Goslar, 38644 Deutschland

**Keywords:** Pre-hospital emergency medicine, Telemedicine, Tele-emergency physician, Emergency medical services, Secondary data analysis, Implementation

## Abstract

**Background:**

Pre-hospital emergency medicine has been facing major challenges for several years due to increasing numbers of emergency calls, limited personnel resources and difficulties in staffing. A tele-emergency physician system provides immediate on-site emergency medical assistance and can support and guide emergency service personnel directly, thereby promoting the optimal use of available resources. Since January 2021, tele-emergency physicians have been deployed as part of a pilot project in the Goslar district in Lower Saxony, Germany. The aim of this study was to conduct a descriptive analysis of changes in on-site emergency physician missions and tele-emergency physician missions between 2021 and 2023.

**Methods:**

To address this research question, a retrospective secondary data analysis of mission protocols was conducted. After data preparation, a descriptive data analysis was performed. Correlation analyses were conducted to compare on-site emergency physician missions and tele-emergency physician missions. Additionally, a technology questionnaire was completed by the tele-emergency physicians after every mission over a period of one and a half years and descriptively analysed to assess connection interruptions during tele-emergency physician missions.

**Results:**

From 2021 to 2023, annual on-site emergency physician missions decreased from 5210 to 3623, and tele-emergency physician missions declined from 1632 to 1066. In terms of mission and treatment durations, there was a statistically significant difference between on-site emergency physician and tele-emergency physician missions across all three years. Between 1 May 2022 and 31 October 2023, 3.3% of tele-emergency physician missions were interrupted.

**Conclusion:**

The findings from this pilot project confirm existing data from other studies and demonstrate that tele-emergency physician systems are an efficient resource in pre-hospital emergency medical services. They relieve emergency physicians in low-priority cases and, after an initial learning curve, from higher-priority cases as well. Furthermore, tele-emergency physicians can be deployed across all diagnostic categories.

**Clinical trial number:**

Not applicable – secondary data analysis.

**Supplementary Information:**

The online version contains supplementary material available at 10.1186/s12873-025-01286-w.

## Background

Pre-hospital emergency medicine in Germany has been facing major challenges for several years. Increasing numbers of emergency calls, limited personnel resources, difficulties in staffing emergency physician positions with qualified emergency physicians, and longer transport distances due to the reduction of alternative care structures are all contributing to the growing strain on emergency medical services [1, 2]. According to statistics from statutory health insurance, the number of emergency cases involving ambulance and on-site emergency physician missions increased from a total of 5.88 million cases in 2012 to 8.15 million cases in 2022 [3, 4]. To maintain a consistent quality of care despite the rising demand, it is essential to optimize the use of available resources and enhance them with supplementary technologies. A tele-emergency physician system provides immediate physician support at the scene and can, by taking over specific cases, offer guidance and direction to on-site emergency personnel [5–9]. Additional applications for tele-emergency physicians can include assisting with secondary transport or providing support in complex emergency situations or major incidents [10, 11]. At the same time, the on-site emergency physician, whose availability is limited, can be allocated to cases where their presence is more medically appropriate [10]. Studies show no significant differences between tele-emergency physicians and on-site emergency physicians in terms of guideline adherence, quality of care or accuracy of diagnosis [12–15]. According to a survey of emergency care experts conducted in 2019, 78% of participants agreed that tele-emergency physician systems should be implemented in every federal state [16]. Since January 2021, a tele-emergency physician system has been tested in the Goslar district, Lower Saxony [17] and was expanded by 2023 to cover about 1.2 million residents across several regions in Lower Saxony. On-site emergency personnel can consult the tele-emergency physician via the dispatch center for specific cases. Communication is done through a smartphone with a high-quality camera, allowing live video and audio—even outside the ambulance. Unlike other systems with fixed cameras, this model uses only mobile devices. Vital signs are transmitted via telemetry, enabling the tele-emergency physician to guide treatment until the patient reaches the hospital.

The aim of this study was to descriptively analyse the changes in on-site emergency physician missions and tele-emergency physician missions between 2021 and 2023after the introduction of the Lower Saxony model:

- How did the number of on-site emergency physician missions and tele-emergency physician missions change during the observation period?
- How did the mission and treatment times of on-site emergency physician missions and tele-emergency physician missions evolve during the observation period?
- To which medical diagnosis categories the on-site emergency physician and tele-emergency physician are called over the three-year period?
- For which levels of medical urgency could the tele-emergency physician be deployed according to the initial NACA score (“National Advisory Committee for Aeronautics“, which is a widely used descriptive tool for assessing the severity of injuries and illnesses in pre-hospital emergency medicine)?
- How often were tele-emergency physician missions terminated, and what reasons led to the subsequent request for an on-site emergency physician?

## Methods

### Study design and setting

To answer the research questions, a secondary data analysis of routine data was conducted using mission protocols (Thieme DokuFORM EPRO-5.1- ABCDE MIND 3.1) for both emergency physician missions and tele-emergency physician missions. In Germany, the organization of emergency medical services (EMS) is regulated at the regional level, resulting in substantial structural variability across federal states and municipalities. Almost every city or district operates its own emergency dispatch centre, which is responsible for call triage and resource deployment. Each dispatch centre follows locally defined protocols, and the Medical Director of Emergency Services (Ärztlicher Leiter Rettungsdienst) determines the criteria for dispatching specific EMS resources. EMS resources typically include ambulances (Rettungswagen, RTW) and emergency physician vehicles (Notarzteinsatzfahrzeuge, NEF). An RTW is usually staffed by two professionals: a paramedic (Notfallsanitäter) and an emergency medical technician (Rettungssanitäter), both of whom are trained in advanced prehospital care. The paramedics undergo a three-year vocational training program covering advanced prehospital care, including airway management, pharmacology, and invasive procedures, and is authorized to perform certain life-saving interventions independently when no physician is available. The emergency medical technician, in contrast, completes a shorter 520-hour program focused on basic life support and patient transport. The NEF carries an emergency physician (Notarzt), who is typically a specialist in anaesthesiology, internal medicine, or surgery with additional prehospital emergency training, and is supported by a paramedic acting as the driver and assistant.

In the context of our project, which includes five municipalities, each operates its own dispatch centre. A single tele-emergency physician located in Goslar provides remote medical support for all participating regions. The tele-emergency physician system has been deployed as part of a pilot project in the Goslar district, Lower Saxony since January 2021 [17]. From July 2021 to September 2023, the deployment area of tele-emergency physicians in Goslar was gradually expanded to include the districts of Northeim, Emsland, Grafschaft Bentheim, and the city and district of Hildesheim, covering a total of nearly 1.2 million residents. Unlike other tele-emergency physician systems, where ambulances are equipped with stationary cameras, the Lower Saxony model uses only a smartphone for audio-video communication. The video transmission is conducted as live streaming during each mission, and the video data is not stored, for privacy reasons. To transmit vital signs to the workstation of the tele-emergency physician during the mission, all ambulances are equipped with a corpuls C3 (defibrillator/monitor with telemetry module, GS Elektromedizinische Geräte, G. Stemple GmbH, Kaufering, Germany). A mobile phone with a high-quality camera, worn as a portable device by the emergency personnel, enables direct communication via video and audio between the emergency personnel, tele-emergency physician, and the patient, even outside the ambulance [17]. Through joint diagnostics and delegation to the on-site emergency personnel, the tele-emergency physician can fully guide the treatment until the patient arrives at the hospital. Thereby, the tele-emergency physician was integrated into the standardized operational procedures of the participating emergency medical services in the areas and is responsible for the assignment with the beginning of the consultation. The on-site emergency personnel alert the tele-emergency physician via the dispatch centre Thereby, the tele-emergency physician was integrated into the standardized operational procedures of the participating emergency medical services in the areas. The on-site emergency personnel alert the tele-emergency physician via the dispatch centre based on defined indications, such as transport refusal (due to a new SOP for the introduction of tele-emergency physician, the tele-emergency physician had to be called for every transport refusal) or analgesia for severe pain. Tracer diagnosis are not an indication for the tele-emergency physician, as due to the need for a rapid response either the highly qualified emergency paramedics or an on-site emergency physician are alerted. In February 2022, the mandatory use of the tele-emergency physician for transport refusals was withdrawn due to a change in the SOP. From June 2023, the SOP for “Analgesia” authorized the administration of fentanyl by emergency paramedics in the district of Goslar. In September 2023, the introduction of the SOP for “Acute chest pain” began the use of the tele-emergency physician for patients with non-life-threatening acute chest pain. The list of indications for NEF missions did not change during the period under review.

### Data sources

**Fig. 1:**
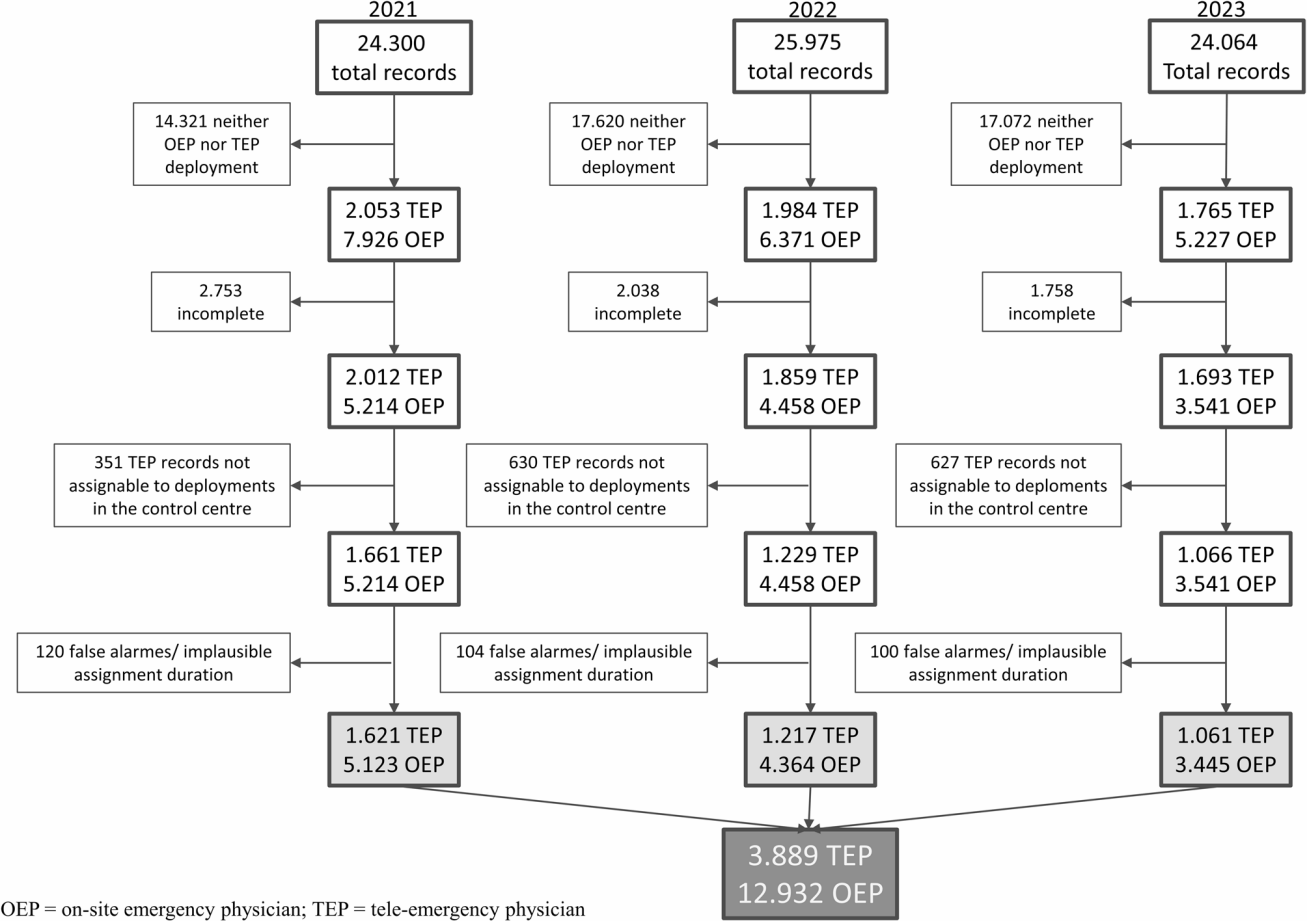
Flow-chart of records

The data source consisted of the mission protocols for all emergency medical service missions coordinated by the Goslar dispatch centre from 2021 to 2023. The mission protocols are standardized protocols that have been authorized by the German Interdisciplinary Association for Intensive Care and Emergency Medicine (Deutsche Interdisziplinäre Vereinigung für Intensiv- und Notfallmedizin, DIVI) and are used for the legal documentation of an emergency mission (DIVI Protocols). The analysis included the following variables from all protocols of on-site emergency physician missions and tele-emergency physician missions in the Goslar district: mission type, mission date, mission duration, treatment duration, diagnosis, and initial NACA score. Due to data protection reasons and since we had only the protocols from the district of Goslar, were the on-site emergency physician is located, it is only possible to distinguish between two types of protocols: on-site emergency physician protocols from Goslar or tele-emergency physician protocols from any of the five regions. Raw data from the years 2021–2023 were available, totalling 74,339 records (Fig. 1). Of these, 19,524 records were marked as on-site emergency physician missions and 5802 as tele-emergency physician missions, including only half-filled duplicates of records, which occur due to the documentation with an e-pen). As the first step, all incomplete records were deleted. A record was defined as complete if the following mandatory fields in the DIVI mission protocol were filled out: sex, respiration, neurological abnormalities and diagnosis. In the second step, the mission numbers in the protocols were compared with the dispatch centre’s records to assess the plausibility of the remaining data after incomplete records had been removed. This comparison showed that more DIVI mission protocols were marked as tele-emergency physician missions than were actually dispatched according to the dispatch centre (e.g. in 2021, 2012 rescue service protocols vs. 1853 deployed missions according to the dispatch centre). Therefore, in the third step, all tele-emergency physician DIVI protocols that could not be matched to a corresponding emergency tele-physician mission number from the dispatch centre were removed from the dataset. In the final step, all missions with an implausible duration of over 4 h or an implausible treatment duration of over 2 h, or those marked as false alarms, were excluded. Ultimately, the dataset included 12,932 emergency physician missions and 3889 tele-emergency physician missions for the data analysis.

Additionally, to the DIVI protocols from 1 May 2022 to 31 October 2023, the tele-emergency physicians filled out a technology questionnaire after each mission (see Appendix). This questionnaire contained multiple-choice questions regarding the quality of the audio and video connection, data transmission, and whether the tele-emergency physician mission was interrupted due to technical issues. Since completing the questionnaire represents an additional effort in everyday work, the questionnaire was only fulfilled for 1.5 years.

### Variable Preparation of the DIVI mission protocols

The variables used for the analysis were prepared as follows: from the mission date, the month and year of the mission were derived. The patient age was recoded from a date variable in the format yyyy: mm into a metric variable. The mission duration was defined as the period between the time of the alert and the time of mission end. The treatment duration was defined as the period between arrival at the scene and the handover of the patient at the destination. For the analysis of diagnoses, a variable was created for each diagnosis group (central nervous system disorders, cardiovascular diseases, respiratory diseases, abdominal disorders, psychiatric disorders, metabolic disorders, gynaecological/obstetric emergencies, other disorders, infections, trauma). These variables indicated whether a diagnosis from the respective group was recorded (yes = 1, no = 0). The diagnoses were categorized according to the Minimal Emergency Dataset (Minimaler Notfalldatensatz, MIND), which contains a defined set of features and descriptions authorized by the DIVI. The variables for sex and NACA scores were used in the analysis without further preparation.

### Data analysis

The data analysis was performed descriptively using IBM SPSS Statistics version 29. To compare on-site emergency physician missions and tele-emergency physician missions, correlation analyses were conducted. Thereby, Pearson correlation for continuous variables (e.g. mission and treatment duration) and Chi²-test for categorial variables (e.g. diagnosis categories). Time series analyses were carried out to analyse the volume of missions over time (either using linear models or ANOVA models to detect fluctuations in specific months). Since this was is a full survey, statistically significant results were interpreted only if a correlation coefficient of > 0.3 was found [18]. The significance level was set at α = 0.05.

The technology questionnaire was analysed descriptively to determine whether there were any disconnections during the tele-emergency physician missions and, if so, the reasons for the disconnection and how the mission was continued. The analysis was carried out using Microsoft Excel.

## Results

**Fig. 2:**
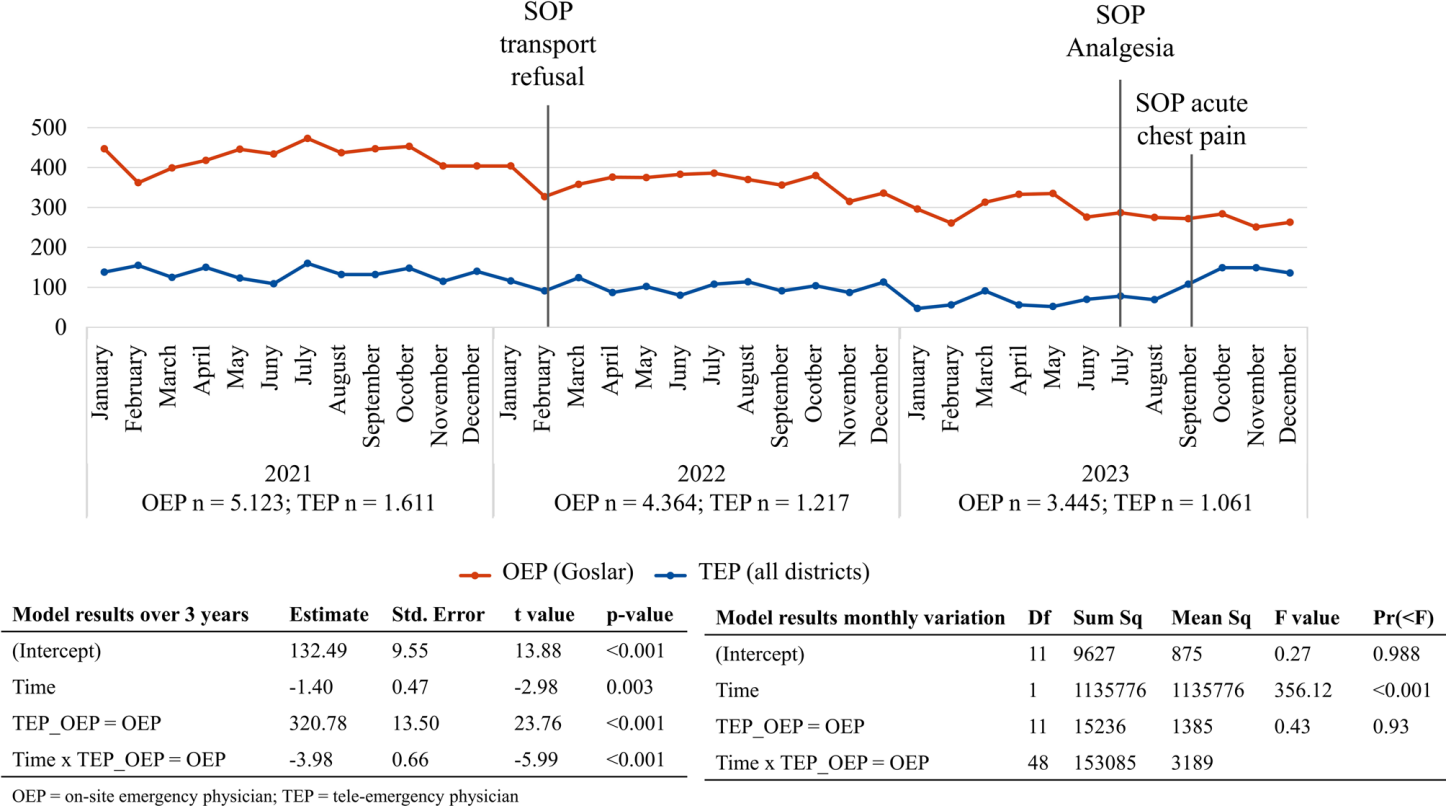
Development of tele-emergency physician and on-site emergency physician mission numbers from 2021–2023

From 2021 to 2023, tele-emergency physician missions decreased from 1627 to 1061 missions per year (Fig. 2). The number of on-site emergency physician missions in the district of Goslar also decreased from 5124 to 3446 missions in 3 years. The annual number of cases differed significantly between tele-emergency and on-site emergency physicians (*p* = 0.021). Over time, tele-emergency physician case counts decreased by approximately 275 cases per year (*p* = 0.042), whereas on-site emergency physician cases started substantially higher and showed a stronger negative trend (interaction β = -564, *p* = 0.021), indicating a steeper decline in on-site emergency physician case numbers. Analysis of monthly case counts revealed a significant overall difference between tele-emergency and on-site emergency physician missions (*p* < 0.001). However, there was no significant seasonal variation in case numbers across months for either group, nor an interaction effect between month and group (both *p* > 0.9), suggesting stable monthly distribution patterns within each group.

**Fig. 3:**
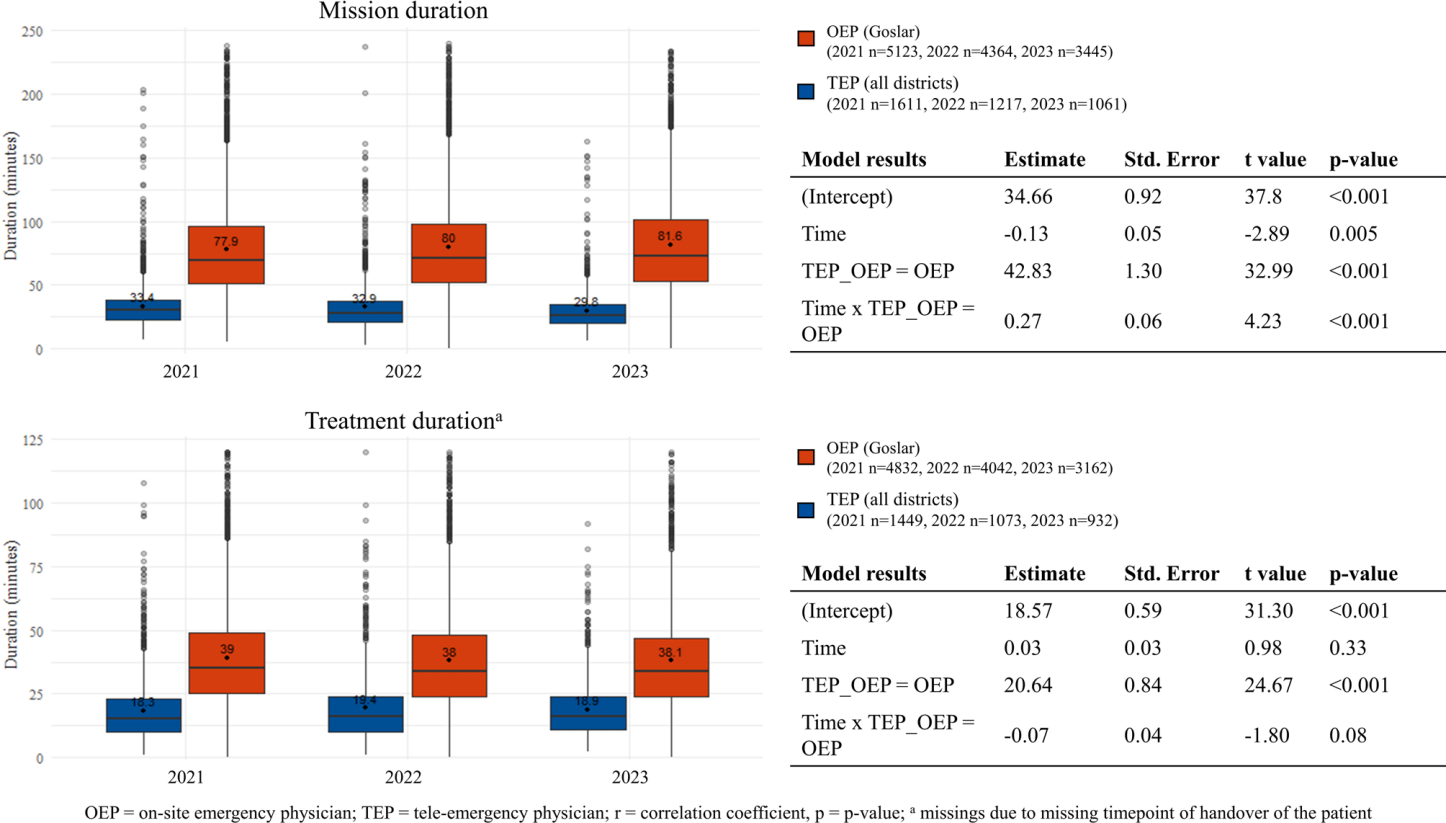
Comparison of mission and treatment times of the tele-emergency physician and the on-site emergency physician from 2021 to 2023

About half of all patients were female (47.9%), and on average the patients were 62 years old (standard deviation 24.7 years). The tele-emergency physician mission duration decreased over the three years from 33.4 min in 2021 to 29.8 min in 2023, while the tele-emergency physician treatment duration remained stable (18.3 min in 2021, 19.4 min in 2022, 18.9 min in 2023) (Fig. 3). The duration of on-site emergency physician missions increased over the years from 77.9 min to 81.6 min, while the duration of treatment also remained constant (39.0 min in 2021, 38.0 min in 2022, 38.1 min in 2023). At baseline, the mean mission duration for tele-emergency physicians was approximately 34.7 min and showed a slight but significant monthly decrease (β = -0.13, *p* = 0.005). On-site emergency physicians had mission durations on average 42.8 min longer at baseline (*p* < 0.001) and exhibited a significantly increasing trend over time compared to tele-emergency physicians (interaction β = 0.27, *p* < 0.001). Baseline treatment time for the tele-emergency physician was about 18.6 min with no significant change over time (β = 0.03, *p* = 0.33). On-site emergency physicians had treatment times 20.6 min longer on average at baseline (*p* < 0.001) and no trend over time was observed.

**Fig. 4:**
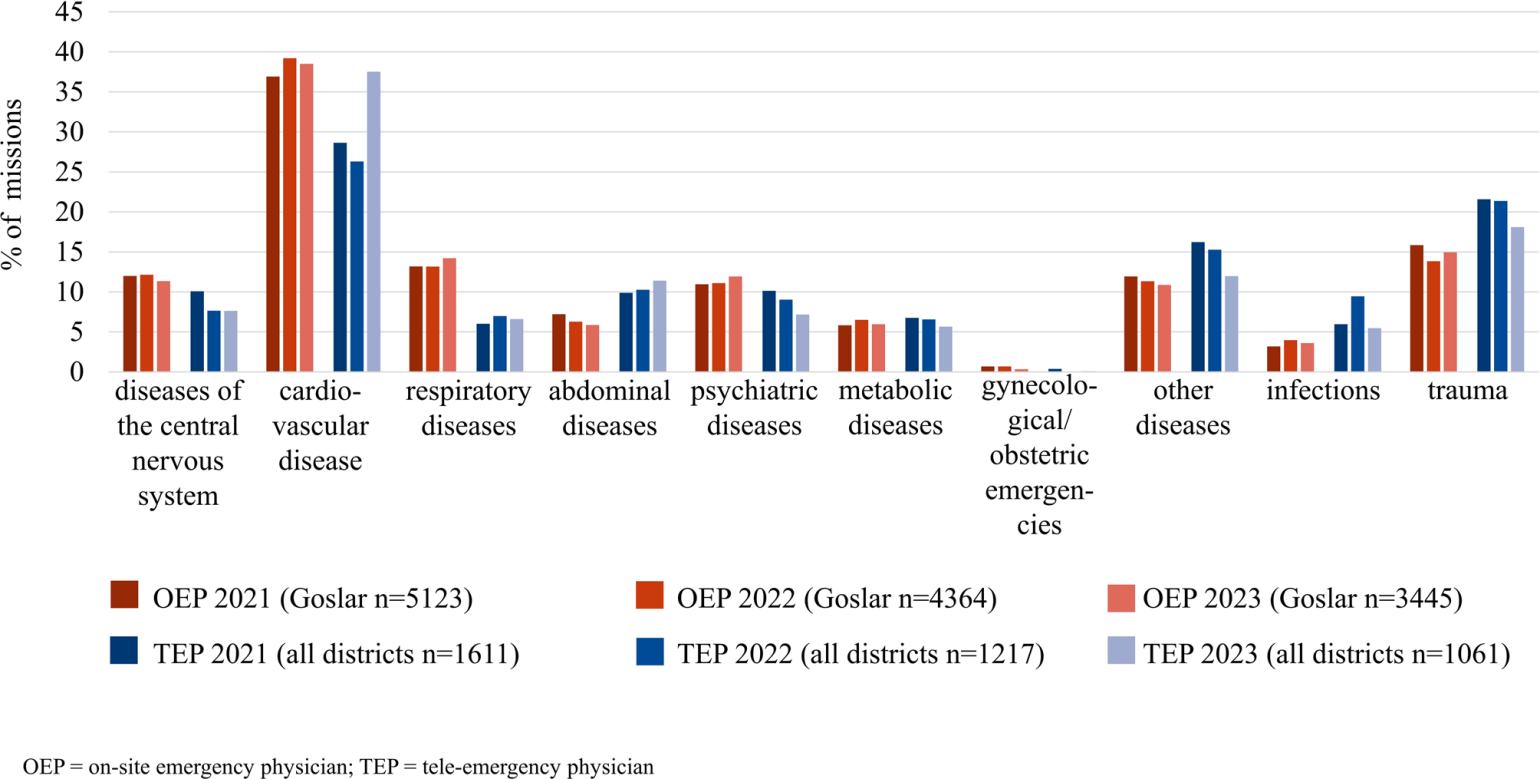
Frequency of diagnosis categories by the tele-emergency physician and the on-site emergency physician mission in the years 2021–2023

The most common diagnoses in both on-site emergency physician missions and tele-emergency physician missions were cardiovascular diseases and trauma, while the least common were gynaecological or obstetric emergencies (Fig. 4). The on-site emergency physician is called out more often to respiratory diseases, whereas the tele-emergency physician is called out more often to trauma and other disorders.

**Fig. 5:**
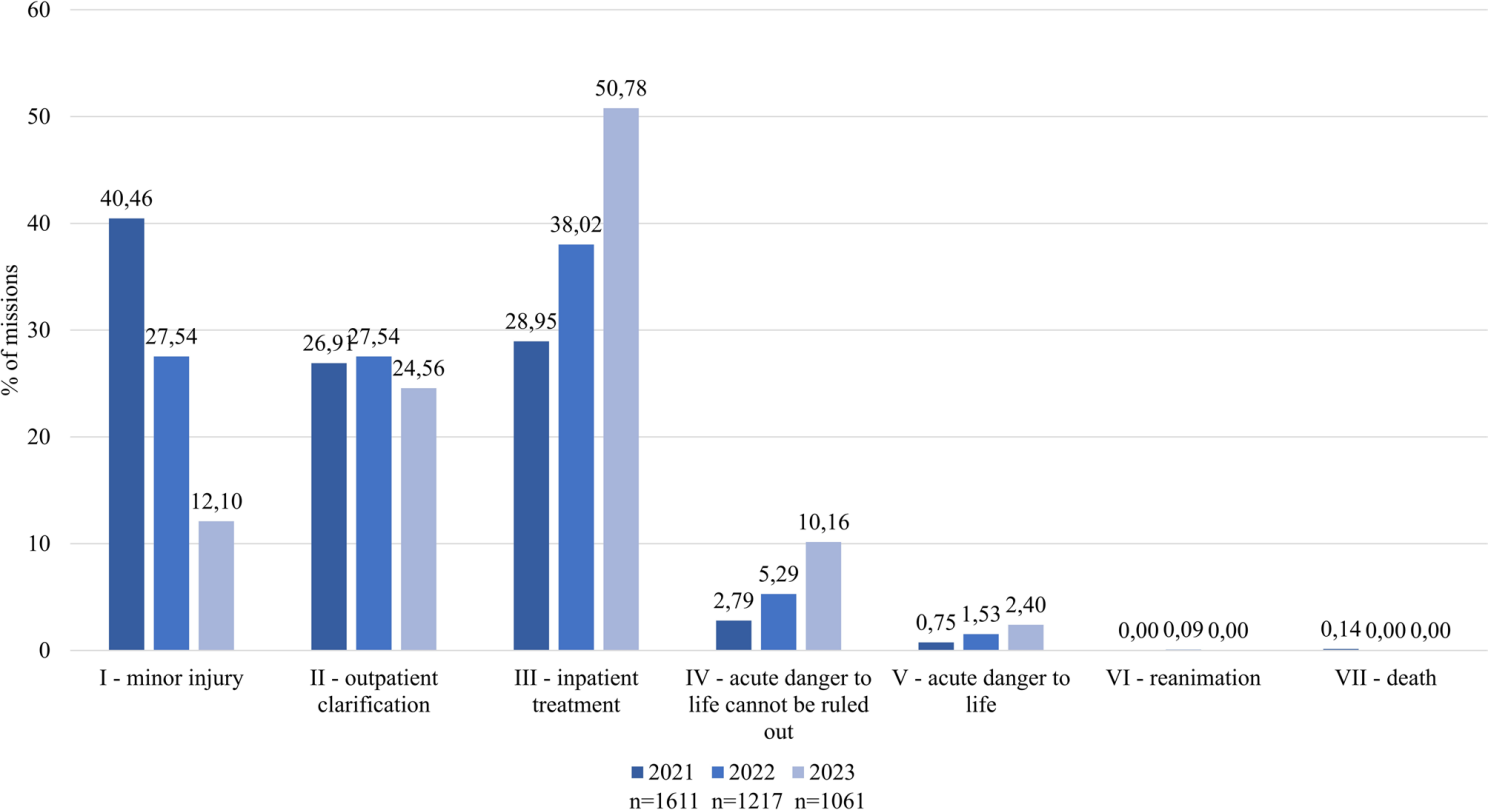
NACA scores of the tele-emergency physician missions from 2021–2023

With regard to the medical urgency of tele-emergency physician missions, it could be observed that the largest proportion of missions with NACA score I was recorded in 2021 (40%), while these decreased in 2022 (28%) and 2023 (12%), and the proportion of missions with NACA score III (29% in 2021; 50% in 2023) and IV (3% in 2021, 10% in 2023) increased (Fig. 5). The proportion of missions with NACA score II remained constant over the three years (25–28%).

In the period 1 May 2022 to 31 October 2023, 61 (3.3%) tele-emergency physician missions were cancelled, 19 of which were continued without medical contact and 42 were treated by an on-site emergency physician (Table 1). In 42 missions, the tele-emergency physician missions were cancelled and transferred to an on-site emergency physician in 20 cases due to medical necessity.

**Table 1:** Results of the technology questionnaire

	*n* = 1828	%
Cancellation of tele-emergency physician assignment	61	3.3
Continuation without medical contact	19	1.0
Additional call for an on-site emergency physician	42	2.3
- No reason known	3	7.1
- Technical fault	19	45.2
- Medical necessity	20	47.6

Reasons for needing medical support: acute dyspnoea (palliative), acute abdomen (syncope during tele-emergency physician assignment), analgesia, anaphylaxis, bradycardia, cardiac arrhythmia, intoxication (patient unable to give consent), N-STEMI, self-harm (language barrier), sepsis (intravenous access difficult), refusal, compulsory admission

Survey-period: 1 May 2022 to 31 October 2023

## Discussion

In this retrospective analysis of DIVI protocols, we were able to show that on-site emergency physician missions decreased by around 30% since the introduction of the tele-emergency physician. This is certainly a multifactorial process. The mission and treatment times of the tele-emergency physician were significantly shorter compared with those of the on-site emergency physician. The comparison of the treated diagnoses shows that the tele-emergency physician could be used for almost all diagnostic categories. Furthermore, we were able to show that the tele-emergency physician was increasingly used for cases with a higher medical urgency (NACA score III to V) during the observation period, but also continued to relieve the burden on ambulance resources for missions in the lower NACA score range. The tele-emergency physician consultation was cancelled in around 3% of the missions.

### Number of missions

The tele-emergency physician system processed 1611 missions in its first year (2021). One reason for this is that it had to be called in for all refusals to transport and in the event of transport being cancelled due to a new SOP for the introduction of tele-emergency medicine. This resulted in regular use and familiarization of all on-site emergency personnel with the technology used. Over the course of the observation period, it can be observed that the numbers of missions for the tele-emergency physician remained relatively constant despite modulation by SOPs (1217 missions in 2022 and 1061 missions in 2023), while the number of missions for the on-site emergency physician decreased continuously during the observation period. One reason for this might be the professionalization of the emergency paramedic job profile in recent years has meant that more rescue service missions can be handled without emergency physicians. Several SOPs were also introduced in the district of Goslar during the period under review, which made it possible to handle missions without an emergency physician, including the release of anaesthetics (narcotics) for analgesia by emergency paramedics followed by the 2023 reform of the Narcotics Drugs Act (Betäubungsmittelgesetz).

Congruently to the observations in Goslar the number of on-site emergency physician missions decreased in Aachen after the introduction of the tele-emergency physician in 2014. At the same time the number of tele-emergency physician missions increased continuously in Aachen [19], while the number of tele-emergency missions in Goslar remained constant over time despite the connection of further municipalities with decreasing numbers of on-site emergency missions. One reason for this might be the withdrawal of the mandatory use of the tele-emergency physician for transport refusals due to a change in the SOP. Only when the districts of Emsland and Grafschaft Bentheim joined (September 2023) was there a significant increase in the number of call-outs. One reason for this might have been that a fixed scheme was used to introduce the tele-emergency physician for the first time: structured training of employees, mandatory test calls and a high level of involvement of the local dispatch centre.

### Mission and treatment times

The mission and treatment times of the tele-emergency physician and the on-site emergency physician differed significantly over the entire treatment period. As other locations have already shown, the tele-emergency physician having no arrival time leads to a significantly shorter mission time (alert to end of mission) compared with the on-site emergency physician [20]. In addition, the tele-emergency physician mission times were also continuously reduced during the observation period despite the increasing medical urgency of the missions according to the NACA score (33 min in 2021 vs. 29 min in 2023). Partly difference might be explained by the fact, that the routine of consultations in cases with low medical urgency, which often involved a refusal of transport or less complexity raised over time. Furthermore, the tele-emergency physician was only consulted after the emergency paramedics had already conducted an initial examination, taken the medical history and carried out initial measures (e.g. setting up IV access). As a result, the tele-emergency physician could concentrate on the core medical activity and withdraw from the mission after a short time.

### Diagnostic categories

The analysis of the diagnostic categories showed that on-site emergency physicians and tele-emergency physicians treated patients in the same diagnostic categories (Fig. 3) which supports findings from previous research [1]. Differences could only be detected in the “respiratory diseases”, “trauma” and “other disorders” categories. The more frequent use of on-site emergency physicians in the “respiratory diseases” category might be ascribed to the mandatory presence of the on-site emergency physician in the event of the need for airway management. The results relating to the “trauma” category showed that the physical presence of the emergency physician in both traumatic and non-traumatic missions requiring analgesia was reduced by calling in the tele-emergency physician. In a multicentre study, Brokmann et al. already found no significant differences in the analgesia of patients with acute coronary syndrome when a tele-emergency physician was called in compared with an on-site emergency physician [15]. The “other disorders” category included a wide range of diagnoses such as acute lumbago, epistaxis and urological conditions, which did not often require a doctor to be present on site either, and could be treated by consulting the tele-emergency physician. The results showed that the tele-emergency physician, like the on-site emergency physician, could be used for almost all diagnostic categories. Nevertheless, it must be noted that the results only provide a superficial overview of the diagnostic categories, each of which can represent very different clinical pictures. A direct comparison of the diagnostic categories of on-site emergency physician and tele-emergency physician is not possible due to the different indications for use alone. In critical emergency situations with a high need for manual skills (e.g. resuscitation) or activities requiring intensive dialogue (e.g. mental illnesses), the deployment of a physical emergency physician was preferable [21]. This also applied to the care of critically ill or injured children.

### NACA score

At the beginning of the observation period, the tele-emergency physician was mainly used for interventions with low urgency (NACA score I and II) and less for imminent or acute danger to life. By 2023, the use of the tele-emergency physician had shifted mainly to NACA III (50%), which included serious but non-life-threatening illnesses that required inpatient care. Studies from Greifswald and Aachen came to similar conclusions: following the introduction of the tele-emergency physician, around half (48% and 57% respectively) of the patients cared for by the tele-emergency physician were categorized with NACA score III [1, 22]. The increase in tele-emergency physician missions with NACA scores IV and V could be explained in the Goslar district by an increasing working routine and – as in other tele-emergency physician locations – primarily by the call-out of the tele-emergency physician for patients in acute mortal danger to bridge the gap until the arrival of the on-site emergency physician or if the on-site emergency physician was unavailable [5, 23]. The tele-emergency physician could also be requested by emergency physicians on a consultative basis. Due to their high level of professional experience and qualifications, they take on a “senior physician function” in the rescue service [5].

### Cancellations

According to the technology questionnaire, around 3% of tele-emergency physician missions were cancelled; in two thirds of these missions, an on-site emergency physician was requested. Technical faults and medical necessity were the most frequently cited reasons for the additional call-out. Medical indications for the additional call-out of an on-site emergency physician were partly critical medical conditions, such as bradycardic cardiac arrhythmia, and partly patients in an exceptional mental state who required compulsory hospitalization. Despite the rural nature of the area of the missions, a possible lack of network coverage did not appear to be a major technological barrier. The future nationwide expansion of the 5G network and the further development of technologies should contribute to a further reduction in technical faults.

## Limitations

For this analysis, only mission protocols that contained a complete 12-digit mission number could be analysed. All missions with a missing or incorrect mission number therefore had to be excluded. Further mission protocols were excluded from the analysis due to incomplete or incorrect documentation. Due to the data quality and incompleteness, but also the retrospective nature of the data collection (some of the data was not collected specifically for this question), the generalizability and validity of the results may be limited. While the examined on-site emergency physician protocols originated only from the district of Goslar, the analysed tele-emergency physician missions took place in all connected districts which is a further limitation of the study, makes it difficult to compare the data and might have an impact on the observed results. A comparison with data before the implementation of the tele-emergency physician was also not possible and no randomization was conducted due to the known methodological and logistical challenges in emergency research [24, 25]. Additionally, the fact that the technical questionnaires do not cover the whole study period are a limitation of our study. Furthermore, this retrospective data analysis did not consider the occurrence of side effects or the diagnostic concordance of the tele-emergency physician with that of the on-site emergency physician or in the hospital. Moreover, with the presented data it was not possible to detect whether the tele-emergency physician was consulted due to the mission itself or due to transport refusal which possibly biases the analysis.

## Conclusion

Between 2021 and 2023, on-site emergency physician missions declined from 5,123 to 3,445, and tele-emergency physician missions from 1,611 to 1,061. Mission and treatment durations differed significantly between both groups across all years. Mission interruptions occurred in 3.3% of tele-emergency cases. Our results confirm previous findings: tele-emergency physicians are a reliable and efficient resource across all urgency levels and diagnoses. Even without video systems, the smartphone-based model in Lower Saxony showed no increase in technical issues.

These findings supported the 2024 amendment to the Lower Saxony Rescue Service Act, (Novelle des Niedersächsischen Rettungsdienstgesetzes) fostering nationwide implementation [26]. Structured integration in new regions is key to long-term use. Future studies should examine patient perspectives as for example already conducted in Greifswald [1], the role of video, and how technical equipment influences care quality. Moreover, the project highlights the ongoing challenge of poor data quality in pre-hospital care and the urgent need for digitalization.

## Electronic supplementary material

Below is the link to the electronic supplementary material.

Supplementary Material 1

## Data Availability

The datasets used and/or analysed during the current study are available from the corresponding author on reasonable request.

## Abbreviations

DIVI: German Interdisciplinary Association for Intensive Care and Emergency Medicine (Deutsche Interdisziplinäre Vereinigung für Intensiv- und Notfallmedizin)
MIND: Minimal Emergency Dataset (Minimaler Notfalldatensatz)
NACA: National Advisory Committee for Aeronautics
