# Supplementary material for: Three years of tele-emergency medicine with mobile on-site audio-video streaming in lower Saxony, Germany – descriptive results of a longitudinal secondary data analysis
Source: BMC Emerg Med. 2025 Jul 15;25:126. doi: 10.1186/s12873-025-01286-w (PMC12261602; doi:10.1186/s12873-025-01286-w)
Supplement: Supplementary file 1 — Supplementary Material 1 [file 12873_2025_1286_MOESM1_ESM.pdf]

# Fragebogen nach einem Einsatz mit TMN

| Einsatzdatum | Einsatznummer | Rettungsmittel |
|---|---|---|
| | | RD GS 87-08-1 |
| | Ausführender NotSan/RettAss | TeleNA |
| | | |

## Technik

- ☐ Problemlos
- ☐ Audioverbindung nicht möglich
  - ☐ Kein Netz
  - ☐ Fehlfunktion Corpuls Mission
  - ☐ Anderes
- ☐ Videoverbindung nicht möglich
  - ☐ Kein Netz
  - ☐ Fehlfunktion Corpuls Mission
  - ☐ Anderes
- ☐ Vitaldatenübertragung nicht möglich
  - ☐ Kein Netz
  - ☐ Fehlfunktion Corpuls Life
  - ☐ Anderes
- ☐ Problemlösung
  - ☐ Einsatz trotzdem möglich durch (Lösung)
  - ☐ Abbruch des TNM Einsatzes
    - ☐ Nachforderung Notarzt
    - ☐ Einsatz ohne weitere ärztliche Einbindung

## Kommunikation im Einsatz

| 0 | 1 | 2 | 3 | 4 | 5 | 6 | 7 | 8 | 9 | 10 |
|---|---|---|---|---|---|---|---|---|---|---|

Sehr schlecht — Sehr gut

## Teamarbeit im Einsatz

| 0 | 1 | 2 | 3 | 4 | 5 | 6 | 7 | 8 | 9 | 10 |
|---|---|---|---|---|---|---|---|---|---|---|

Sehr schlecht — Sehr gut
